# Challenges in the Contemporary Management of Syphilis among Pregnant Women in New Orleans, LA

**DOI:** 10.1155/2019/2613962

**Published:** 2019-02-13

**Authors:** Irene A. Stafford, Alexandra Berra, Charles G. Minard, Virginia Fontenot, Rachel H. Kopkin, Eliza Rodrigue, Charles M. Roitsch, Martha W. Rac, James B. Hill

**Affiliations:** ^1^Baylor College of Medicine/Texas Children's Hospital, Department of Obstetrics and Gynecology, Houston, TX, USA; ^2^Louisiana State University Health Sciences Center, Department of Obstetrics and Gynecology, New Orleans, LA, USA; ^3^Baylor College of Medicine/Texas Children's Hospital, Institute for Clinical and Translational Research, USA; ^4^Baylor College of Medicine/Texas Children's Hospital, Section of Neonatology, Houston, TX, USA; ^5^Baylor College of Medicine/Texas Children's Hospital, Department of Obstetrics and Gynecology, San Antonio, TX, USA

## Abstract

**Objective:**

The aim of this retrospective review is to evaluate trends in the management of maternal and congenital syphilis (CS) in a tertiary care center in New Orleans, LA.

**Study Design:**

All cases of maternal and neonatal syphilis over a five year period at Touro Infirmary, New Orleans, LA, were identified using ICD-9/10 codes. Charts were reviewed for demographic and obstetrical variables, stage of syphilis at diagnosis, lab values, and treatment regimen. Newborn treatment and other outcomes were recorded.

**Results:**

During the study period 106 infected mother-baby pairs were identified. Of these, 73 charts are available for review. 41% (n = 30) of women received inadequate therapy according to their stage of disease. 9% of newborns (n = 6) were found to be symptomatic for CS; however, only 83.3% of these were admitted to the neonatal intensive care unit. Only 20% (n = 6) of infants were adequately treated with an extended penicillin regimen if the mother was not adequately treated. Furthermore, only 63.0% of newborns had a nontreponemal titer performed.

**Conclusion:**

With rising rates of CS, strict adherence to the 2015 CDC guidelines for treatment of syphilis must be maintained.

## 1. Background

Syphilis is a genital ulcerative disease caused by the bacterium* Treponema pallidum *that progresses to systemic disease over time when left untreated. With the advent of syphilotherapy and global initiatives directed at eradicating infection worldwide, rates of primary and secondary syphilis reached their nadir in 2001 and 2002 at 2.1/100,000 in the United States, with complete elimination in some countries [1–5]. Historically, the group most affected was men who engage in sexual activities with men, but in recent years, there has been an increase in cases attributed to a growing number of infected women in all regions of the United States and among all ethnic and age groups [6]. In the United States, during 2013–2017, the primary and secondary syphilis rate among women more than doubled (increased 155.6%). The rate of infections in pregnant women and their newborns has also peaked with over 918 congenital cases reported in the United States during 2017, representing a 153.3% increase relative to 2013 (Center for Disease Control and Prevention [CDC] Sexually transmitted disease surveillance, 2017). The complications associated with maternal syphilis infection are widespread and include congenital infection [7–9] ultimately leading to possible fetal compromise, preterm birth, polyhydramnios, and fetal death. The pathophysiology of in utero fetal infection parallels maternal disease with early stages of syphilis among mothers posing the highest fetal infectivity risk. There is a 50% risk of fetal infection with maternal primary syphilis increasing to 83% with early latent disease [10, 11]. Disease progression in the fetus has been well described and includes early hepatomegaly and placentomegaly followed by amniotic fluid infection and fetal hematologic abnormalities with progression to hydrops fetalis. Detailed ultrasound of the infected fetus can detect these findings and is currently part of the recommended diagnosis and treatment algorithm for antenatal management of syphilis [8–10, 12–14]. Neonatal complications are well described and involve intensive care management with likely resultant long term sequelae for the developing child [15–17]. True risk factors for treatment failure for both the pregnant woman and her fetus are multifactorial and include delayed diagnosis, delayed or inadequate treatment (<30 days prior to delivery) and advanced stage of congenital syphilis as evident by sonographic fetal abnormalities. With early detection and appropriate syphilotherapy, successful treatment can be anticipated in fetuses with sonographic evidence of early disease [7–9]. Among high risk communities, Louisiana ranks first in the nation for congenital syphilis, with a rate of CS in 2016 nearly four times the national rates at 94/100,000 [6].

Increasing national rates of syphilis among pregnant women and their newborns may involve problems with patient compliance, provider adherence to the currently established CDC 2015 sexually transmitted infections guidelines, failure to detect disease prior to delivery, or a combination of these factors [6].

The aim of this retrospective review is to evaluate trends in the obstetrical and neonatal management of syphilis in a major tertiary care center in New Orleans, Louisiana, to elucidate factors impacting high rates of CS in Louisiana and inform improvement efforts.

## 2. Methods

After institutional review board approval from Louisiana State University Health Sciences Center, all cases of deliveries complicated by maternal syphilis from January 1, 2013, to December 31, 2017, at Touro Infirmary, New Orleans, LA, were identified using ICD-9 and 10 codes. Women with reported serofast syphilis were excluded. Maternal charts were reviewed for demographic and obstetrical variables, stage of syphilis at diagnosis, testing patterns, nontreponemal titer values, treatment regimen during pregnancy, sonographic findings, and interval from treatment to delivery. Newborn information including birth weight, Apgar score, neonatal intensive care admission, physical and radiologic exam findings, nontreponemal titers, treatment regimen, and other outcomes was recorded. Patient demographics and clinical characteristics were summarized by means with standard deviations, medians with minimum and maximum values, or frequencies with percentages. Chi square was used to determine differences in demographic variables and stage of disease for women with and without correct treatment regimens.

## 3. Results

Between January 1, 2013, and December 31, 2017, 16,926 deliveries occurred at Touro Infirmary, New Orleans, LA. One hundred and six parturient diagnosed with syphilis during the pregnancy and their infants were identified. Of these, 30 were serofast as identified by provider documentation and 3 had incomplete medical records leaving 73 mother and newborn charts available for review. The number of maternal syphilis cases increased yearly with 6 cases in 2013, to 26 in 2017 with rates of maternal infection of 1.6/1000 in 2013 to 7.95/1000 in 2017. The demographics of the population are listed in Table 1. The majority (80.8%) of women were diagnosed with late latent or unknown syphilis infection (n = 59). The mean gestational age at diagnosis was 16 weeks (Table 1). Only 43 women (58.9%) received the correct benzathine penicillin regimen according to the 2015 CDC STI Treatment guidelines given the stage of disease, regardless of interval of time between treatment and delivery. Although all women had third trimester and intrapartum testing, ten women (13.6%) were diagnosed in the third trimester with late presentation to care. Two women were diagnosed with new infection through repeat testing in the third trimester and treatment was initiated. Only 3 women in the cohort were diagnosed during the intrapartum period and had no care or complete treatment prior to delivery. There was no significant difference in rates of prenatal care or stage of disease between groups that were and were not treated correctly (p value = .372, .771). The majority of records (98.6%) did not document any health department notification or confirmation of treatment. Nineteen percent of women admitted to using illicit substances during pregnancy, with cannabis being the most commonly used at 14.5%, followed by cocaine at 4.1% and benzodiazepines and opiates at 1.4% each. Coinfection with Human Immunodeficiency Virus-type 1 was found in one patient (1.3%) and rates of* Neisseria gonorrhea *and* Chlamydia trachomatis* coinfection were found in 8.2 and 9.6% of patients, respectively (Table 2).

The mean gestational age at delivery was 37.6 weeks and most women (60%) underwent an uncomplicated vaginal birth. Of the thirteen women with preterm births, only two (15.3%) delivered prior to 30 days from completed treatment. Two stillbirths occurred among the infected cohort (2.7%).  Table 3 describes labor and delivery characteristics of the study population.

Neonatal data is listed in Table 4. Mean birth weight was 3018 ± 569 gm and 58% of infants were male. Only 15% of fetuses underwent antenatal sonographic evaluation for CS and one had findings consistent with congenital infection as evident by elevated middle cerebral artery velocities prior to treatment. Twelve newborns were diagnosed as highly likely to have CS and treated with full dose ten day penicillin (CDC Congenital Syphilis Guidelines, 2015). Nine percent of newborns were symptomatic at birth; however only 83.3% of the symptomatic newborns were admitted to the neonatal intensive care unit. Only 20% (n = 6/30) of infants were adequately treated with either single dose or an extended penicillin regimen if the mother was not adequately treated. Furthermore, only 63.0% of exposed newborns had a nontreponemal titer performed. Twenty-six percent (n = 16) of asymptomatic newborns considered less likely or unlikely to have congenital syphilis according to the CDC congenital syphilis treatment guidelines received single dose penicillin. Infected newborns were diagnosed based on found to have abnormal lumbar puncture results (15.1%), hematologic abnormalities (12.3%), neuroimaging and ophthalmic abnormalities (4.1%), and extremity problems (pseudoparalysis) (2.7%) (Table 5).

## 4. Conclusions

The aim of this five-year retrospective study was to analyze specific patient factors and provider practices that may have contributed to the escalating rate of syphilis in women and their newborns in a large obstetrical center in New Orleans, LA, in order to identify missed opportunities for treatment and evaluate specific barriers, deficiencies, and quality measures that require attention. Although Louisiana reports the highest rate of congenital syphilis in the United States [6], rates of infection have increased among all regions of the country, in every age group and among all race/ethnic groups [6]. California reported the fifth consecutive year for increases in the number of infants born with congenital syphilis along with a 7-fold increase in early infection among women between 2012 and 2017 (cdph.ca.gov). International data reveal similar trends. From 2010 to 2015, the rate of infectious syphilis in Canada increased by 85.6%, from 5.0 to 9.3 cases per 100,000 population [18]

Patient factors, including the escalating opioid misuse and the addiction epidemic, have contributed to increased rates of syphilis and other sexually acquired infections as risky sexual practices may accompany drug seeking behavior [19, 20]. In 2017, the Oklahoma State Department of Health reported one of the largest outbreaks of syphilis involving over 80 residents in several months linked to a methamphetamine and opiate ring (Ok.gov/health2/documents/table/summary/states). As societal norms change, social media channels for contemporary dating, including anonymous location based applications designed for mutually interested persons, make tracking reportable infections impossible. Lack of access to regular prenatal care contributes to adverse perinatal outcomes for untreated and undiagnosed diseases including syphilis. Twenty-one percent of CS cases were attributed to this cause in current surveillance data [21]. In our cohort, 11% attended less than 3 prenatal care visits, and of those who did, the mean gestational age at initiation into care was 14 weeks with treatment initiated soon thereafter. Only 2 (4.6%) of the women who received inadequate therapy had no prenatal care, which is not significantly different than those who did receive adequate treatment (p value = .372). Lack of education about sexual health, along with high rates of illiteracy, poverty, and homelessness, makes testing and treatment unachievable in many urban settings, including New Orleans. The Centers for Disease Control and Prevention have dedicated resources to state and local health departments in high prevalence states including Texas, Louisiana, California, Illinois, Florida, Georgia, Maryland, and Ohio to strengthen congenital syphilis activities and initiatives.

Clinical practice patterns for infection in pregnant women have also been of concern. In a recent MMWR addressing increasing rates of congenital syphilis during the 2014 year, 458 maternal charts were reviewed and revealed that approximately 43% of women with syphilis seeking prenatal care did not receive treatment. Of the women that were treated, 30% received inadequate treatment [21]. Our results reveal similar missed opportunities for successful treatment in our cohort of pregnant women. We report that 58.9% of women received inadequate therapy according to their stage of disease, with 27% of these cases involving either erroneous dosage or incorrect medication interval. Although errors in medication interval adherence by the patient may have contributed to this, it is difficult to confirm through this retrospective chart review where interviews about compliance were not recorded, or if erroneous dosing was related to clinical practice errors. Although the 2015 CDC STI guidelines recommend that pregnant women with early syphilis should be treated with a single dose of penicillin G, these guidelines also support consideration of an additional dose one week after as there is evidence to suggest benefit for CS prevention [7, 14]. In our analysis, only 33% of women received a second dose for early disease. Current national recommendations also support detailed sonographic fetal evaluation for congenital syphilis as fetuses with abnormal findings indicate higher treatment failures [7, 14]. In our analysis, only 15% of infected mothers with care underwent a detailed ultrasound to interrogate the fetus for findings consistent with congenital syphilis. Similar to our findings concerning provider practices, a large study evaluating intrauterine fetal demise found only 34% of providers tested for syphilis as an etiology, demonstrating a lack of consistent testing and management for this significant cause of fetal death [22]. Of note, testing for prenatal syphilis is not a requirement in six states, despite the current CDC, American Academy of Pediatrics (AAP), and American College of Obstetricians and Gynecologists (ACOG) recommended screening guidelines stating that all pregnant women should undergo testing at the first prenatal visit, at third trimester, and at delivery if at high risk, a guideline implemented in Louisiana based delivery centers (Warren HP 2018, CSC.gov/std/tg2015/screening-recommendations [23, 24]). Due to escalating rates of congenital syphilis, the United States Preventive Service Task force recently released recommendations that all pregnant women should be tested for syphilis as early as possible to allow for time sensitive interventions that may prevent congenital disease [24, 25].

Of greater concern are the missed opportunities to test and diagnose infected asymptomatic newborns. The likelihood of congenital syphilis among newborns exposed to maternal infection during gestation but who received adequate treatment has a normal physical examination and a low or nonreactive nontreponemal serologic test for syphilis is not fully known [26] as long term data evaluating persistent infection in early childhood are lacking and the nontreponemal test may falsely exclude newborns with disease [26]. In addition, current recommendations state that single dose benzathine penicillin administered to the asymptomatic newborn with a serum quantitative nontreponemal serologic titer equal to or less than fourfold the maternal titer* is optional*, further complicating the decision tree for the provider handling a newborn whose mother is considered treated with either one or two possibly or even three doses of benzathine penicillin [14]. Our cohort revealed that 26% (n = 16) of asymptomatic newborns considered low risk for congenital syphilis received single dose penicillin; however only 63.0% of the newborns had a nontreponemal titer performed, despite current recommendations to screen all at-risk infants. Of the available newborn charts reviewed, only 20% of infants were appropriately treated with an extended penicillin regimen if the mother was not adequately treated after detailed chart review. The lack of appropriate treatment in the remaining 80% involved unverified or nonvalidated treatment plans miscommunicated on the chart.

The strengths of this study include that this detailed chart review of all cases of maternal and neonatal syphilis was recorded and analyzed entirely from a clinical perspective without the use of reportable public health data that may not collect this level of clinical detail. Although this remains a systems quality challenge within a hospital system in New Orleans, it may reflect similar issues in many other cities and states experiencing the growing syphilis epidemic. The largest weakness of this study, consistent with other research involving CS, involves the lack of long term data on the newborns who were either partially treated or presumed unlikely to have syphilis, those who did and did not receive single dose penicillin, and those with and without sonographic evidence of disease. Future studies addressing these issues may clarify these questions and streamline the many options surrounding the neonatal provider faced with a newborn who might be at risk and the maternal fetal medicine physician asked to counsel an infected mother about the future of her child. Although this was a large five year analysis, it remains limited by retrospective methods of data collection at a single site.

Consistent with national surveillance data, our findings suggest significant pitfalls in antenatal diagnosis and treatment of syphilis in pregnant women and their newborns. Variation in screening laws and nonadherence to national guidelines along with vague treatment algorithms for the asymptomatic newborn considered low risk based on often unverified clinical data has undoubtedly contributed. Universal implementation of early and repeated serologic screening may prevent some cases of congenital syphilis; however, attention to improvement efforts such as provider education, patient outreach, adherence to treatment guidelines, and vigilant follow-up is necessary to ensure treatment success for this preventable disease.

## Figures and Tables

**Table 1:** Maternal demographics and syphilis characteristics: N = 73.

Age (years):	25.2 ± 6.2
Gravidity:	2.9 ± 2.0
Parity:	2.5 ± 1.6

Race:	
(i) African American	65 (90.3)
(ii) White	3, (4.2)
(iii) Hispanic	4, (5.6)
(iv) Other	1, (1.4%)

Complete Prenatal Care (>3 visits)	65, (89.0)

Number of Prenatal Visits:	8.8 ± 4.3

Gestational Age at First Prenatal Visit (weeks):	14.1 ± 7.6

Prior Preterm Birth:	10, (13.7)

Substance Abuse:	14, (19.4)

*Syphilis Characteristics*	

Stage of Syphilis	
Primary	10, (13.7)
Secondary	3, (4.1)
Early Latent	1, (1.4)
Late Latent/unknown	59, (80.1)

Gestational Age at Diagnosis (Weeks):	16.9 ± 10.6

Titer at Diagnosis, n = 70	
1:4 or less	32, (45.7)
1:8 to 1:32	28, (40%
1:64 or greater	10, (14.3)

Year of Diagnosis	
2013	6, (8.2)
2014	10, (13.7)
2015	13, (17.8)
2016	18, (24.7)
2017	26, (35.6)

Health Department Notified of Syphilis Diagnosis/treatment confirmed	1, (1.4)

PCN allergic	6, (8.2)
Desensitized	6 (100%)

Correct Penicillin Administration *∗∗*	43, (58.9)
Primary	5, (11.6)
Secondary	2, (4.7)
Early Latent	1, (2.3)
Late Latent or unknown	35, (81.4)

Incorrect treatment *∗∗*	30, (41)
Primary	3, (10.0)
Secondary	1, (3.3)
Early Latent	0
Late Latent or unknown	26, (86.6)

Data are n, (%) or mean SD.

*∗∗*p value = .771 for difference in correct/incorrect treatment according to stage of disease.

**Table 2:** Antepartum variables: N = 73 unless otherwise specified.

Variable	Yes (%)
Cerclage	1, (1.4)

GDM or Pre-GDM	1, (1.4)

Chronic Hypertension	4, (5.6)

Thyroid Disease	0, (0)

Asthma	9, (12.3)

Hepatitis B	1/72 (1.4)

Hepatitis C	0, (0)

HIV	1, (1.4)
*∗*Patient was taking antiretrovirals	

Gonorrhea	6, (8.2)
*∗*All patients were treated	

Chlamydia	7, (9.6)
*∗*87.5% documented treated	

Antenatal Corticosteroid Administration	2/70 (2.9)

Fetus Interrogated Sonographically for Evidence of CS	11/72 (15.3)

Of those interrogated, Evidence of CS Present	1/11 (9.1)

Oligohydramnios	2/70 (2.9)

Intrauterine Growth Restriction	6/71 (8.45)

Data are n (%).

**(a):** 

GA at Delivery (weeks): mean ± SD	37.6 ± 3.57

**(b):** 

Variable	Yes
Spontaneous Vaginal Delivery	42/70 (60.0)

Operative Vaginal Delivery	4/70 (5.7)

Cesarean Delivery	24/70 (34.3)

Preterm Delivery	13/69 (18.9)

PPROM	6/70 (8.6)

Magnesium Administration	3/70 (4.3)

Chorioamnionitis	5/70 (7.1)

Intrauterine Fetal Demise	2/70 (2.8)

Data are n (%).

**Table 4:** Neonatal variables: N = 73 unless otherwise specified.

Birth weight (grams)	3018 ± 569

Gender	
Female	29/69, (42.0)
Male	40/69, (58.0)

1 Minute Apgar(mode):	9

5 Minute Apgar(mode):	9

Symptomatic at Birth:	6, (8.2)

VDRL/RPR Performed	46, (63.0)

Titer results ( n = 46)	
Nonreactive	22, (47.8)
1:1	4, (8.70)
1:2	5, (10.9)
1:4	5, (10.9)
1:8	4, (8.70)
1:16	3, (6.5)
1:32	2, (4.4)
Note: One was “reactive” with no value.	

NICU Admission	17, (23.3)

Average NICU Stay (days)	10.7 ± .5

Diagnosed as highly likely to have congenital syphilis	12, (16.4)

Treated with extended PCN regimen when mother not adequately treated (n = 30)	6, (20.0)

Asymptomatic low risk newborn treated with single dose PCN (n = 61)	16, (26.2)

Data are n (%) or mean ± SD.

**Table 5:** Neonatal signs and symptoms: N = 73.

Variable	Yes
Abnormal Neuroimaging	1, (1.4)
*∗*Neuroimaging performed on 12 subjects	

Abnormal Ophthalmic Exam	2,(2.7)

Jaundice	6, (8.2)

Non-Immune Hydrops	0, (0)

Rhinitis	0, (0)

Hepatosplenomegaly	0, (0)

Rash	0, (0)

Extremity Problems (pseudoparalysis)	2, (2.7)

Skeletal Survey Done	7, (9.60)

Blood Transfusion Required	1, (1.4)

Intraventricular Hemorrhage	0, (0)

Bronchopulmonary Dysplasia	0, (0)

Retinopathy of Prematurity	0, (0)

Necrotizing Enterocolitis	1, (1.4)

Aneuploidy	0, (0)

Supplemental Oxygen Requirement	7, (9.6)

Ventilator Support Required	1, (1.4)

Surfactant Administered	1, (1.4)

Sepsis Workup Performed	25, (34.2)
*∗*4% positive	

Neonatal Anomaly	9, (12.3)

Lab Abnormalities	9, (12.3)

Abnormal Lumbar Puncture	11, (15.1)

Intrauterine fetal demise	2, (2.7)

Data are n (%).

## Data Availability

All deidentified data can be formally requested via email to the Principal Investigator, Irene Stafford, MD.
